# Unveiling coding of prediction and sharpening of perceptual features through multivariate pattern analysis

**DOI:** 10.1177/23982128231200722

**Published:** 2023-09-21

**Authors:** Sung-Mu Lee, Petar Raykov, Andrea Greve

**Affiliations:** MRC Cognition and Brain Sciences Unit, University of Cambridge, Cambridge, UK

**Keywords:** Predictive coding, sharpening, perception, repetition, fMRI multivariate pattern

## Abstract

A recent study by Blank, Alink and Büchel, uses multivariate neuroimaging to investigate how the human brain processes the strength of face-related expectations and explores whether these expectations are represented in the same regions that process facial stimuli. In line with predictive coding theories, their study presents compelling evidence that the brain adjusts its processing based on the certainty of expectations. This occurs exclusively within high-level face-sensitive regions, rather than across the entire processing hierarchy. Here we critically discuss these findings and outline potential directions for future research to better understand how the human brain expects, processes, and perceives images.

## Introduction

When we navigate our environment, we have preconceived expectations of what or who we will likely encounter. However, these expectations are not all equal; they carry varying degrees of likelihood based on our past experiences, that is, we are likely to expect a colleague’s face at our workplace but a family member’s face at home. A recent study by Blank et al. (2023) explores how the human brain handles the strength of these face-related expectations and whether they are represented in the same regions that code for the expected content itself, that is, facial stimuli. In line with predictive coding (PC) theories, which suggest perception is a hierarchical process, their findings provide first compelling evidence that the brain adapts its processing of anticipated faces based on how certain those expectations are, but only within high-level face-sensitive regions and not throughout the entire processing hierarchy. Here, we will discuss the significance and implications of their findings for future research, such as separating analyses for strong and weak predictions, in order to test contrasting theories, examining the impact of mental imagery on representational patterns, and exploring the connectivity between brain regions.

## Methods and findings

Blank et al. (2023) utilised functional resonance imaging (fMRI) in conjunction with sophisticated multivariate techniques to address the question of how expectation strength and content are coded. Participants were presented with scene cues, which they used to predict the identities of faces with different probabilities: low (10%), intermediate (30%) or high (60%). To test whether the strength of face expectations can be identified in addition to the predicted identity of the faces, the authors tested three models by which similarity of multivariate patterns before and after acquiring predictions might change with prediction strength: (a) similarity gradually increases with expectation (graded response), (b) similarity increases only for highly expected faces (high response) and (c) similarity increases for both unexpected and highly expected faces (U-shape response), indicating that multiple processes might take place, that is processing predicted faces and prediction error responses.

Their data revealed that only high-level face-sensitive regions, such as the anterior temporal lobe (aTL), coded for the representation of expected faces, both the graded and high response models were significant, while the u-shape model was not. By contrast lower-level face-sensitive regions like occipital face area (OFA) and fusiform face area (FFA) did not code for expected faces. This result confirms that when scene priors that evoke face expectations (but do not contain images of faces themselves) are presented, predictions and their strength are indeed selectively represented in higher-level regions instead of the entire processing hierarchy.

Once faces were actually encountered, only the OFA revealed expectation-dependent coding, again both the graded and high response models were significant. This increase in similarity of representation pattern for presented faces which were highly expected was interpreted by the authors as sharpening in perceptual features, consistent with sharpening theory.

Finally, univariate analyses for presented faces uncovered a U-shape pattern in FFA, showing increased activity for both highly expected and unexpected faces. This observation was interpreted to suggest the simultaneous occurrence of two processes in lower-level face-sensitive regions: enhanced processing of highly expected faces and heightened processing of prediction errors for unexpected faces.

## Importance

According to PC theory, human brains constantly generate predictions about forthcoming experiences. This PC theory has gained substantial influence in explaining various cognitive processes such as perception, learning, and memory (Barron et al., 2020; Friston, 2018; Greve et al., 2017). Previous studies have shown that anticipated sensory inputs trigger pre-activation within the sensory cortex (Kok et al., 2014, 2017) less clear, however, is how the strength of expectations is represented in the brain. It is possible that expectation strength is explicitly coded at all levels of the processing hierarchy. However, by utilising a novel task, Blank et al. (2023) offer new empirical evidence suggesting that higher-level brain regions encompass sensory predictions and also selectively represent the strength of these predictions. Furthermore, the study provides evidence for the sharpening of multivariate fMRI patterns during image repetition. Interestingly, the results also suggest that the brain efficiently processes sensory information by focusing specifically on unexpected stimuli at lower levels of the processing hierarchy. These findings carry significant implications for understanding how the brain processes repeated stimuli while simultaneously being guided by prior expectations.

## Discussion

While acknowledging the limitations of small effect sizes and the use of right-hemisphere-restricted regions of interest, as discussed in the paper, we present several potential avenues for future directions.

First, the authors found increased similarity of multivariate patterns in OFA for observed faces that were highly expected, which was interpreted as sharpened facial features. According to sharpening theory, neural activity becomes more selective to preferred features after image repetition, resulting in increased similarity in representations of the repeated image. However, it is worth noting that more similar representations can also be accounted for by PC theory, which explains the activation through two distinct neural populations (Friston, 2005): (a) neurons encoding predictions provide a more precise sensory signal, leading to increased similarity in their multivariate pattern and (b) neurons encoding prediction errors represent the mismatch between sensory evidence and predictions, resulting in decreased similarity in representations. Therefore, to differentiate PC and sharpening theories, one could test the unique prediction from PC theory, that is, the presence of prediction error units.

One possibility involves separating the similarity matrix for highly and lowly expected faces. According to PC theory, lowly expected faces generate greater prediction errors, leading to greater activity and pattern similarity for lowly expected faces than highly expected faces. Directly testing this prediction can be accomplished by using the data presented in this paper or through future studies. Another way to test prediction error units is to measure laminar responses with high spatial resolution fMRI, for example, at 7 T. According to PC theory, decreased activity and pattern similarity for repeated/expected images are represented by prediction error units which are located at superficial layers.

The second consideration, which has also been acknowledged by the authors, is the potential impact of participants engaging in mental imagery of highly expected faces during the 4- to 8-second interval between cues (scenes) and targets (faces). This mental imagery process could significantly influence the representational patterns of scenes, activating mental representations of faces. Consequently, this might affect the similarity between the presented faces in the localiser scan and the scene presentations in the test sessions. Moreover, participants may exhibit varying degrees of engagement in mental imagery, leading to increased variability of activation patterns within their brains. An intriguing avenue for future studies is to investigate whether our brains automatically generate predictions during all stages of perception and if so, when this predictive process occurs. Techniques with high temporal resolution, such as electroencephalography (EEG) and magnetoencephalography (MEG), offer the potential to answer this question.

Another promising direction for future research is to explore PC theory in terms of the connectivity between brain regions. Although the authors have demonstrated that higher-level regions encode predictive information, it would be valuable to investigate whether and how this information is propagated from higher-level to lower-level regions. To accomplish this, future studies could employ multivariate connectivity measures such as informational connectivity (Anzellotti and Coutanche, 2018), dynamic causal modelling (Friston et al., 2003), or network coding models (Ito et al., 2020) to assess how predictive information is transformed and transmitted between face-selective regions, such as aTL and FFA/OFA, during face-processing tasks.

In summary, the study conducted by Blank et al. (2023) makes a significant contribution to the growing body of evidence supporting PC theories. More importantly, this study demonstrates for the first time that the strength of expectation is selectively represented in higher-level brain regions, which also code expected content. However, this study also raises some outstanding questions, particularly concerning the roles of PC and sharpening in perceiving a repeated image. Addressing these questions in future research would provide further insight into the underlying mechanisms that support different levels of predictions in the human brain.
